# Engineered Dual‐Function Antibody‐Like Proteins to Combat SARS‐CoV‐2‐Induced Immune Dysregulation and Inflammation

**DOI:** 10.1002/advs.202504690

**Published:** 2025-07-06

**Authors:** Yizhuo Wang, Chenwu Bai, Kerui Hou, Zhen Zhang, Ming Guo, Xin Wang, Fan Yang, Xin Liu

**Affiliations:** ^1^ School of Pharmaceutical Sciences Zhongnan Hospital of Wuhan University Wuhan University Wuhan 430071 China; ^2^ Departmant of Radiology, Union Hospital Tongji Medical College Huazhong University of Science and Technology Wuhan 430021 China; ^3^ Institute for Vaccine Research Animal Bio‐Safety Level III Laboratory / Center for Animal Experiment Wuhan University School of Medicine Wuhan 430071 China; ^4^ State Key Laboratory of Virology and Biosafety Modern Virology Research Center and RNA Institute, College of Life Sciences Wuhan University Wuhan 430071 China

**Keywords:** antibody‐like protein, immune dysregulation, mannose‐binding lectin, SARS‐CoV‐2, spike protein

## Abstract

Complement dysregulation and immune hyperactivation are pivotal factors contributing to the mortality associated with SARS‐CoV‐2 infection. Engineered Antibody‐like proteins (ALPs) targeting the SARS‐CoV‐2 spike protein are engineered to address immune dysregulation in COVID‐19. In this study, Lectifitin‐36 and Lectifitin‐41, two such ALPs, are developed using cDNA display technology. These ALPs demonstrate strong binding affinity for the spike protein and effectively inhibit its interaction with ACE2 and several C‐type lectins, including MBL, DC‐SIGN, and L‐SIGN. Both in vitro and in vivo analyses reveal that Lectifitin‐36 and Lectifitin‐41 suppress complement activation via the lectin pathway, reduce neutrophil extracellular trap (NET) formation, and attenuate hyper‐inflammatory responses. In mouse models, Lectifitin‐36 and Lectifitin‐41 significantly mitigate inflammation, NETosis, and lung tissue damage induced by the spike protein. These results suggest that these ALPs hold promise as therapeutic candidates for alleviating SARS‐CoV‐2‐induced immune dysfunction, with the potential to reduce severe COVID‐19 outcomes and long‐term sequelae. This study underscores the therapeutic potential of targeting spike protein‐mediated immune modulation as an innovative approach to combat COVID‐19.

## Graphical abstract

Engineered antibody‐like proteins block the SARS‐CoV‐2 spike protein from binding to host receptors and suppress immune overactivation through an MBL‐based scaffold. These dual‐function proteins effectively alleviate inflammation, complement activation, and lung injury, offering a promising therapeutic strategy against severe COVID‐19 and its complications.

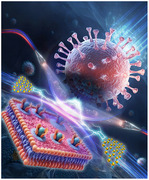

## 1 Introduction

During SARS‐CoV‐2 infection, the spike (S) protein expressed on the viral surface, along with S protein embedded in extracellular vesicles (EVs) released by infected cells, interacts with the host's humoral immunity‐associated pattern recognition molecules (PRMs) and cellular immunity‐linked pattern recognition receptors (PRRs).^[^ ^1^ ^]^ This interaction drives a persistent dysregulation of immune responses, including complement over‐activation, excessive formation of neutrophil extracellular traps (NETs), and hyper‐inflammation.^[^ ^2^ ^]^ These dysregulated responses precipitate cytokine storm syndromes, organ dysfunction, acute lung injury (ALI), acute respiratory distress syndrome (ARDS), thrombosis, and multi‐organ damage, significantly increasing mortality in COVID‐19 patients.^[^ ^3^ ^]^ Furthermore, these immune pathologies contribute to the emergence of long‐term symptoms collectively termed “long COVID,” or post‐acute sequelae of SARS‐CoV‐2 infection (PASC).^[^ ^4^ ^]^

Aberrant complement activation exacerbates cytokine‐driven hyper‐inflammation and thrombotic microangiopathy, playing a pivotal role in the pathogenesis of diseases such as ALI.^[^ ^5^ ^]^ Specifically, SARS‐CoV‐2 S protein binds PRMs such as mannan‐binding lectin (MBL), initiating lectin pathway (LP)‐mediated complement activation.^[^ ^6^ ^]^ This process amplifies systemic inflammation by recruiting neutrophils, monocytes, and macrophages while promoting the release of cytokines and chemokine.^[^ ^7^ ^]^ Concurrently, endothelial damage and platelet activation trigger intravascular coagulation, further compounding tissue damage.^[^ ^8^ ^]^ Therapeutic strategies targeting excessive complement activation have shown promise in mitigating severe COVID‐19 outcomes.^[^ ^9^ ^]^ For instance, Narsoplimab, an antibody targeting MASP‐2 (MBL‐associated serine protease‐2), has demonstrated efficacy in blocking LP‐mediated complement activation in critically ill COVID‐19 patients.^[^ ^10^ ^]^ Another candidate, HG4, a humanized antibody targeting MASP‐2, effectively inhibits LP activation at nanomolar concentrations, underscoring the therapeutic potential of complement‐targeted interventions.^[^ ^11^ ^]^

Neutrophils play a critical role in SARS‐CoV‐2‐induced immune dysregulation.^[^ ^12^ ^]^ The interaction of angiotensin‐converting enzyme 2 (ACE2) and C‐type lectins on neutrophils with the SARS‐CoV‐2 spike protein activates neutrophils, triggering the release of factors such as genomic DNA, histones, myeloperoxidase (MPO), neutrophil elastase (NE), citrullinated histone 3 (CitH3), and cathepsin G (CG), leading to the formation of neutrophil extracellular traps (NETs).^[^ ^13^ ^]^ Excessive NET formation exacerbates immunothrombosis, tissue damage, and multi‐organ dysfunction, contributing to the severe progression of COVID‐19.^[^ ^14^ ^]^ Inhibition of ACE2‐ and C‐type lectin‐S protein interactions has been proposed as an effective strategy to prevent pathogenic NETosis.^[^ ^15^ ^]^ Notably, CLEC2, a spleen tyrosine kinase‐coupled C‐type lectin, directly binds the receptor‐binding domain (RBD) of the S protein, inducing NETosis and platelet activation. Experimental CLEC2‐Fc inhibitors have shown potential in suppressing NET formation and thromboinflammation, reducing the risk of severe progression and long‐term sequelae of SARS‐CoV‐2 infection.^[^ ^16^ ^]^

Hyper‐inflammation represents the central mechanism underlying severe tissue and organ damage in SARS‐CoV‐2 infection.^[^ ^17^ ^]^ Myeloid cells such as monocytes, macrophages, and dendritic cells are central to this inflammatory cascade. Lu *et al.* demonstrated that co‐culturing SARS‐CoV‐2 with myeloid cells induces the release of high levels of cytokines and chemokines, indicating activation of these cells and an exaggerated inflammatory response.^[^ ^18^ ^]^ Further analysis revealed that in COVID‐19 patients with hyperinflammation, myeloid cells express C‐type lectins such as DC‐SIGN, L‐SIGN, LSECtin, ASGR1, and CLEC10A. It showed that the SARS‐CoV‐2 spike protein binds to these lectins, aggravating the inflammatory response. Moreover, nanobodies targeting the spike protein were found to block both ACE2‐mediated viral entry and the pro‐inflammatory response driven by the spike‐lectin interaction. These results suggest that inhibiting the binding of the SARS‐CoV‐2 spike protein to C‐type lectins on myeloid cells could provide a potential strategy to mitigate excessive inflammation in COVID‐19.

**Figure 1:**
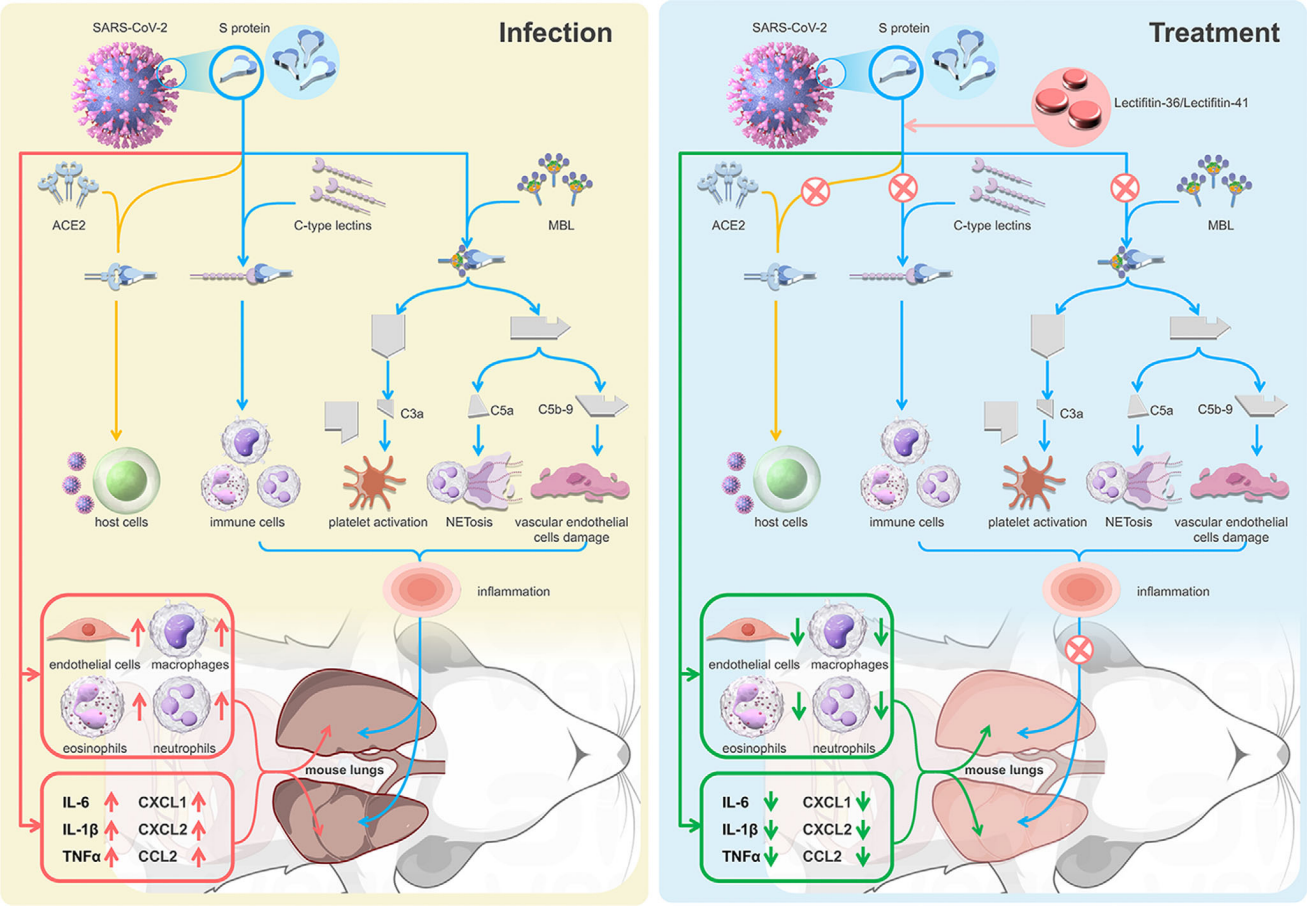
Mechanisms of Targeted Therapy with Lectifitin‐36 and Lectifitin‐41. This figure illustrates the pathogenesis of SARS‐CoV‐2 infection and the therapeutic potential of Lectifitin‐36 and Lectifitin‐41. On the left, SARS‐CoV‐2 spike (S) protein binds to host cell receptors ACE2 and C‐type lectins, triggering complement activation through the lectin pathway. This leads to the production of inflammatory mediators such as C3a and C5a, which activate platelets, induce NETosis, and damage vascular endothelial cells, exacerbating inflammation and lung injury. On the right, Lectifitin‐36 and Lectifitin‐41, antibody‐like proteins (ALPs), block the S protein's interaction with ACE2 and C‐type lectins, inhibiting complement activation and inflammation. This reduces cytokine release, immune cell recruitment, and lung damage in mouse models, highlighting their therapeutic potential in mitigating SARS‐CoV‐2‐induced immune dysfunction. ACE2: Angiotensin‐converting enzyme 2; MBL: Mannose Binding Lectin; NETosis: Neutrophil extracellular trap formation contributing to inflammation; IL: Interleukin; TNF‐α: Tumor necrosis factor‐alpha.

In this study, we engineered two high‐affinity antibody‐like proteins (ALPs), Lectifitin‐36 and Lectifitin‐41, leveraging the carbohydrate recognition domain of MBL and an ACE2‐mimicking peptide as a backbone. Using cDNA display technology, these ALPs were optimized to bind the S protein with high specificity.^[^ ^19^ ^]^ Lectifitin‐36 and Lectifitin‐41 competitively inhibit S protein interactions with ACE2 and C‐type lectins, effectively blocking neutrophil activation, platelet aggregation, and lectin pathway complement activation. These ALPs demonstrated potent inhibition of SARS‐CoV‐2‐induced hyper‐inflammation and NETosis, positioning them as promising candidates for therapeutic intervention in COVID‐19 and related conditions (**Figure** **1**).

## 2 Results

### 2.1 Engineering and Isolation of ALPs Targeting the SARS‐CoV‐2 Spike Protein

To generate antibody‐like proteins (ALPs) that specifically target the SARS‐CoV‐2 S protein, we utilized recombinant MBL and ACE2 mimetic peptides as backbone proteins. Using the receptor‐binding domain (RBD) of the S protein as bait, we conducted four rounds of cDNA display. For the final two rounds, alternating selection between the RBD and the S protein was performed. Using enzyme‐linked immunosorbent assay (ELISA), we screened 195 clones and identified two with high binding affinities to both the RBD and the S protein: Lectifitin‐36 and Lectifitin‐41 (Figure S1a, Supporting Information). These clones were confirmed as candidates and subsequently expressed in *E. coli*. Purification was achieved via Ni‐NTA affinity chromatography, yielding the target proteins Lectifitin‐36 and Lectifitin‐41 (Figure S1b, Supporting Information).

Lectifitin‐36 and Lectifitin‐41 exhibited potent binding activity to the RBD of the S protein, with EC_50_ values in the low nanomolar range (**Table** **1**; Figure S2a, Supporting Information). Notably, both ALPs showed even stronger binding affinities for the full‐length S protein compared to the RBD. Surface plasmon resonance (SPR) analysis further validated these interactions, revealing sub‐nanomolar dissociation constants (K^D^) of 0.96 and 1.18 nm for Lectifitin‐36 and Lectifitin‐41, respectively (Figure S2b, Supporting Information). These results indicate that Lectifitin‐36 and Lectifitin‐41 exhibit strong binding affinities to the SARS‐CoV‐2 S protein.

**Table 1:** Binding activity of Lectifitin‐36 and Lectifitin‐41 against Spike protein (EC_50_, nm).

Group	Target	EC_50_[nm]
Lectifitin‐36	RBD	3.251
Lectifitin‐36	S	2.709
Lectifitin‐41	RBD	2.825
Lectifitin‐41	S	2.287

### 2.2 Lectifitin‐36/Lectifitin‐41 Inhibits Interactions Between SARS‐CoV‐2 Spike Protein, ACE2, and C‐Type Lectins

**Figure 2:**
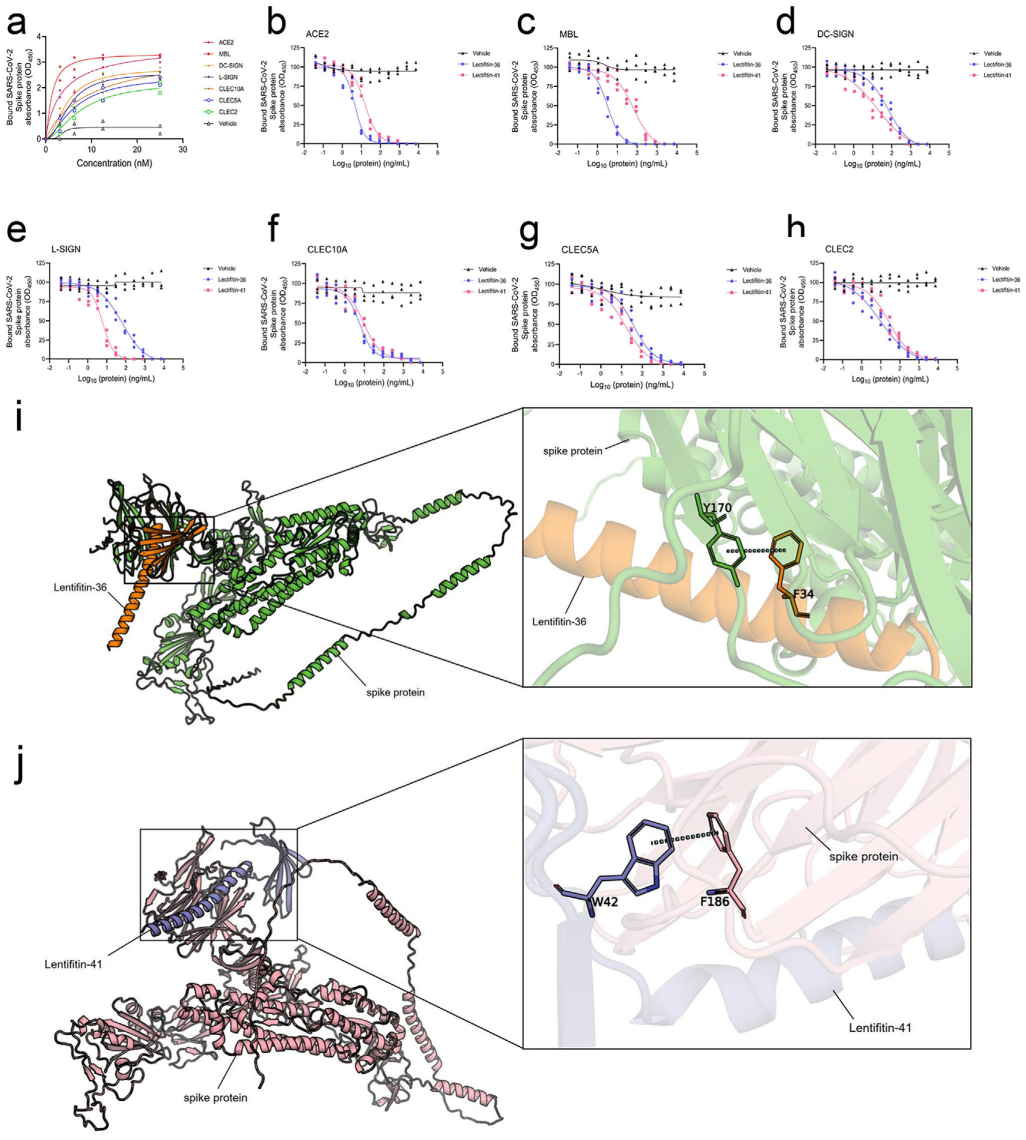
Interaction analysis of SARS‐CoV‐2 spike protein, ACE2, C‐type lectins, and Lectifitin‐36/Lectifitin‐41. a) ELISA dose‐response curves showing the binding of SARS‐CoV‐2 S protein to ACE2 and various C‐type lectins. b‐h) Competitive ELISA analysis of Lectifitin‐36 and Lectifitin‐41 inhibiting S protein interactions with ACE2 and the C‐type lectins. Data represent mean ± SEM from two or three independent experiments. (*n*=3). i) Simulated 3D structure of the Lectifitin‐36/SARS‐CoV‐2 spike protein complex. (Left) The SARS‐CoV‐2 spike protein is shown in green, Lectifitin‐36 in orange, with the local refinement region highlighted by a black box. (Right) Detailed interaction interface after local refinement: bound residues are represented as sticks (green for spike protein; orange for Lectifitin‐36). Predicted π‐π stacking interactions are indicated by dashed lines. j) Simulated 3D structure of the Lectifitin‐41/SARS‐CoV‐2 spike protein complex. (Left) The SARS‐CoV‐2 spike protein is colored pink, Lectifitin‐41 in purple, with the local refinement region marked by a black box. (Right) Detailed interaction interface after local refinement: bound residues are displayed as sticks (pink for spike protein; purple for Lectifitin‐41). Predicted π‐π stacking interactions are represented with dashed lines.

ACE2 and various C‐type lectins, abundantly expressed on immune cells such as neutrophils, monocytes, and macrophages, mediate pro‐inflammatory responses when interacting with the SARS‐CoV‐2 spike protein. These responses are closely associated with disease severity. To evaluate the ability of Lectifitin‐36/Lectifitin‐41 to inhibit these interactions, we first investigated the binding activity of the S protein to ACE2 and several C‐type lectins (MBL, DC‐SIGN, L‐SIGN, CLEC5A, CLEC10A, and CLEC2) using ELISA. The spike protein showed dose‐dependent binding to ACE2 with high affinity (EC_50_ in the low nanomolar range). Similarly, dose‐dependent binding was observed for all tested C‐type lectins, with EC_50_ values spanning the nanomolar range (**Figure** **2**a and **Table** **2**).

**Table 2:** Binding activity of spike protein to ACE2 and C‐type lectin receptors (EC_50_, nm).

Receptor	EC_50_[nm]
ACE2	3.858
MBL	1.306
DC‐SIGN	4.593
L‐SIGN	5.154
CLEC5A	6.405
CLEC10A	9.748
CLEC2	7.686

We next performed competitive ELISA to investigate the inhibitory capacity of Lectifitin‐36/Lectifitin‐41 on these interactions. Lectifitin‐36 and Lectifitin‐41 effectively blocked spike binding to ACE2 with IC_50_ values in the low nanogram per milliliter range (Figure 2b and **Table** **3**).

**Table 3:** Inhibitory activity of ALPs against S‐receptor binding (IC_50_, nm).

Receptor	Lectifitin‐36	Lectifitin‐41
ACE2	4.625	15.22
MBL	3.211	64.68
DC‐SIGN	62.86	16.47
L‐SIGN	65.21	6.173
CLEC5A	37.61	16.05
CLEC10A	5.786	9.238
CLEC2	9.601	26.63

This inhibitory effect extended to the spike protein's interactions with MBL and other C‐type lectins. Both ALPs demonstrated broad‐spectrum inhibition against MBL, DC‐SIGN, L‐SIGN, CLEC5A, CLEC10A, and CLEC2. Lectifitin‐36 exhibited significantly higher potency (IC_50_ <10 ng mL^−1^) against MBL, CLEC10A, and CLEC2 compared to other targets, whereas Lectifitin‐41 showed distinct inhibitory profiles, achieving sub‐10 ng mL^−1^ IC_50_ values for L‐SIGN and CLEC10A (Figure 2c–h and Table 3).

These results suggest that Lectifitin‐36 and Lectifitin‐41 are not only capable of blocking the interaction between S and ACE2, but also inhibit binding to multiple C‐type lectins in a dose‐dependent manner.

To investigate the interactions between Lectifitin‐36/Lectifitin‐41 and the SARS‐CoV‐2 Spike protein, researchers predicted their binding conformations through molecular docking simulations. The results revealed that in Lectifitin‐36, the F34 residue anchors the ligand's aromatic ring via π‐π stacking interactions through its benzene ring, while the Y170 hydroxyl group may form a hydrogen‐bonding network. Lectifitin‐36 could induce conformational rearrangements in the S protein, causing local β‐sheet twisting. In Lectifitin‐41, the upper pyridine ring engages in face‐to‐face π‐π stacking with the indole ring of W42, while the lower fluorophenyl group participates in edge‐to‐face π interactions with the benzene ring of F186. These interactions may facilitate the transition of the receptor‐binding domain (RBD) from the “up” conformation to the “down” conformation (**Figure** 2i‐j).

### 2.3 Lectifitin‐36/Lectifitin‐41 Inhibits SARS‐CoV‐2 Spike‐Induced Complement Activation via the Lectin Pathway

Upon SARS‐CoV‐2 infection, mannose‐binding lectin (MBL) binds to the spike protein, triggering complement activation via the lectin pathway. This activation converts complement factors C3 and C5 into C3a, C5a, and C5b‐9. C3a activates platelets, C5a triggers neutrophil extracellular trap (NET) formation, and C5b‐9 damages vascular endothelial cells, leading to thromboinflammation.^[^ ^6a^ ^]^ To investigate whether Lectifitin‐36 and Lectifitin‐41, derived from MBL, interfere with MBL‐S protein interaction and inhibit lectin pathway activation, we evaluated complement deposition (C3a, C5a, C5b‐9) under different conditions.

**Figure 3:**
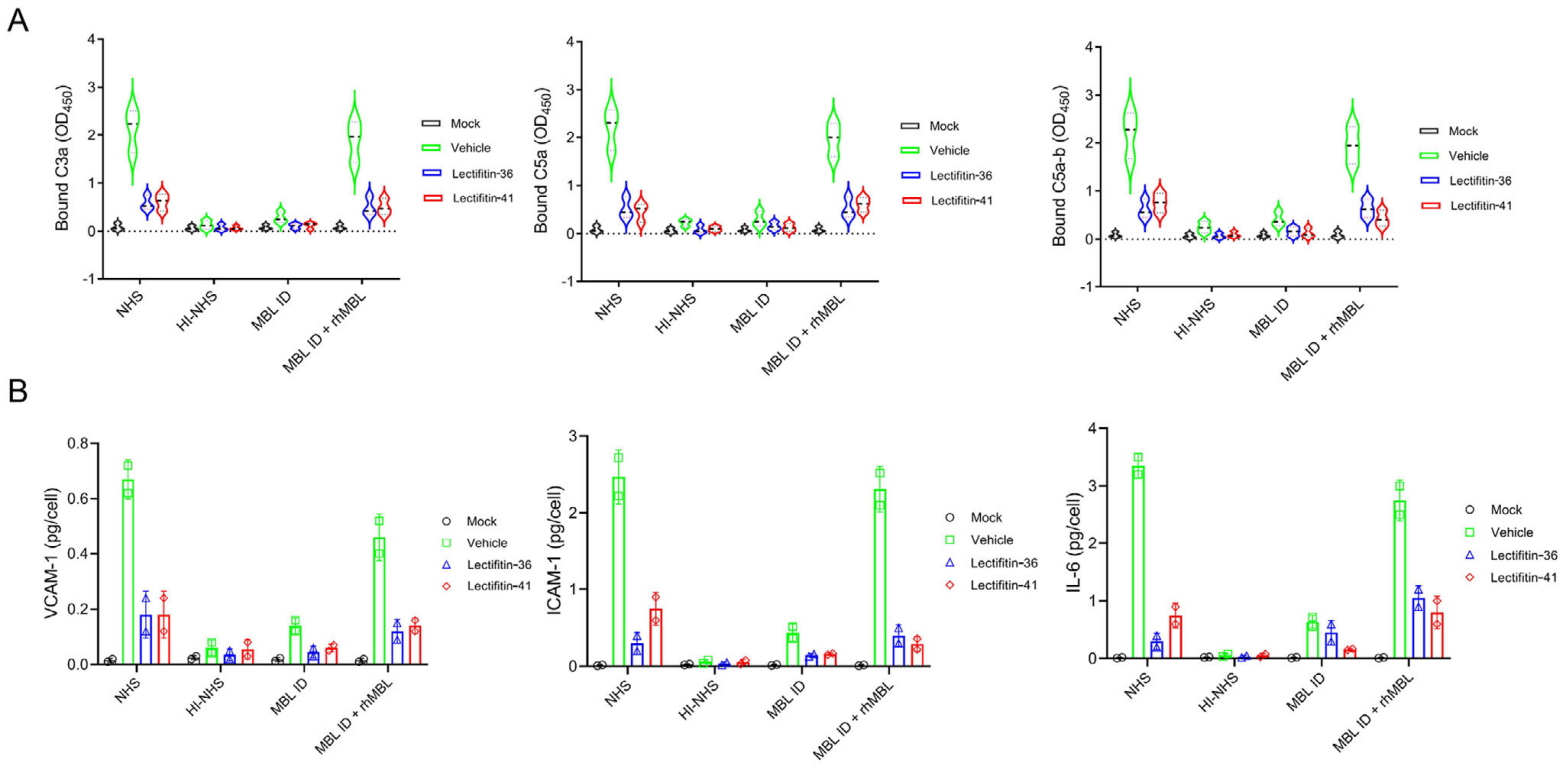
Inhibition of SARS‐CoV‐2 spike protein‐induced complement activation by Lectifitin‐36 and Lectifitin‐41. A) ELISA dose‐response curves for complement activation markers (C3a, C5a, and C5b‐9) in the presence of Lectifitin‐36 and Lectifitin‐41. Complement activation was evaluated in normal human serum (NHS), MBL‐depleted serum, and serum reconstituted with recombinant MBL, after incubation with SARS‐CoV‐2 S protein. B) Data showing the levels of inflammatory biomarkers (VCAM‐1, ICAM‐1, and IL‐6) in response to the inhibition of complement deposition by Lectifitin‐36 and Lectifitin‐41 under various serum conditions, including normal human serum (NHS), heat‐inactivated NHS, and MBL‐depleted serum. Each bar represents mean ± SEM (*n* = 3).

Lectifitin‐36, Lectifitin‐41, or bovine serum albumin (BSA) was incubated with normal human serum (NHS), heat‐inactivated NHS (HI‐NHS), MBL‐depleted serum (MBL‐D), or MBL‐D reconstituted with recombinant human MBL (MBL‐D + rhMBL), then added to SARS‐CoV‐2 spike protein‐coated plates. After incubation, complement activation markers were quantified by ELISA. BSA with NHS served as a control. Complement activation occurred in NHS‐treated samples, with HI‐NHS and MBL‐depleted conditions validating the specificity of the effects (**Figure** **3**).

As shown in Figure 3a, Lectifitin‐36 and Lectifitin‐41 blocked MBL binding to the S protein, preventing complement activation and deposition. BSA with NHS induced significant complement deposition. In HI‐NHS, no complement deposition was detected. When MBL‐depleted serum was reconstituted with rhMBL, complement deposition was restored, confirming that spike‐induced activation occurs via MBL. These results demonstrate that Lectifitin‐36 and Lectifitin‐41 blocked MBL‐S protein interaction and inhibited lectin pathway activation.

As shown in Figure 3b, inflammatory biomarkers (VCAM‐1, ICAM‐1, and IL‐6) were measured to assess the inhibition of spike‐induced inflammation by Lectifitin‐36 and Lectifitin‐41. Lectifitin‐36 and Lectifitin‐41 significantly reduced VCAM‐1, ICAM‐1, and IL‐6 levels in NHS compared to BSA, with a dose‐dependent effect. No reduction was seen in HI‐NHS or MBL‐depleted serum, confirming the lectin pathway dependency. These findings suggest that Lectifitin‐36 and Lectifitin‐41 blocked SARS‐CoV‐2 spike protein interactions with MBL, preventing lectin pathway activation and inflammation.

### 2.4 Lectifitin‐36/Lectifitin‐41 Blocks SARS‐CoV‐2 Spike‐Induced NETosis in Mice

**Figure 4:**
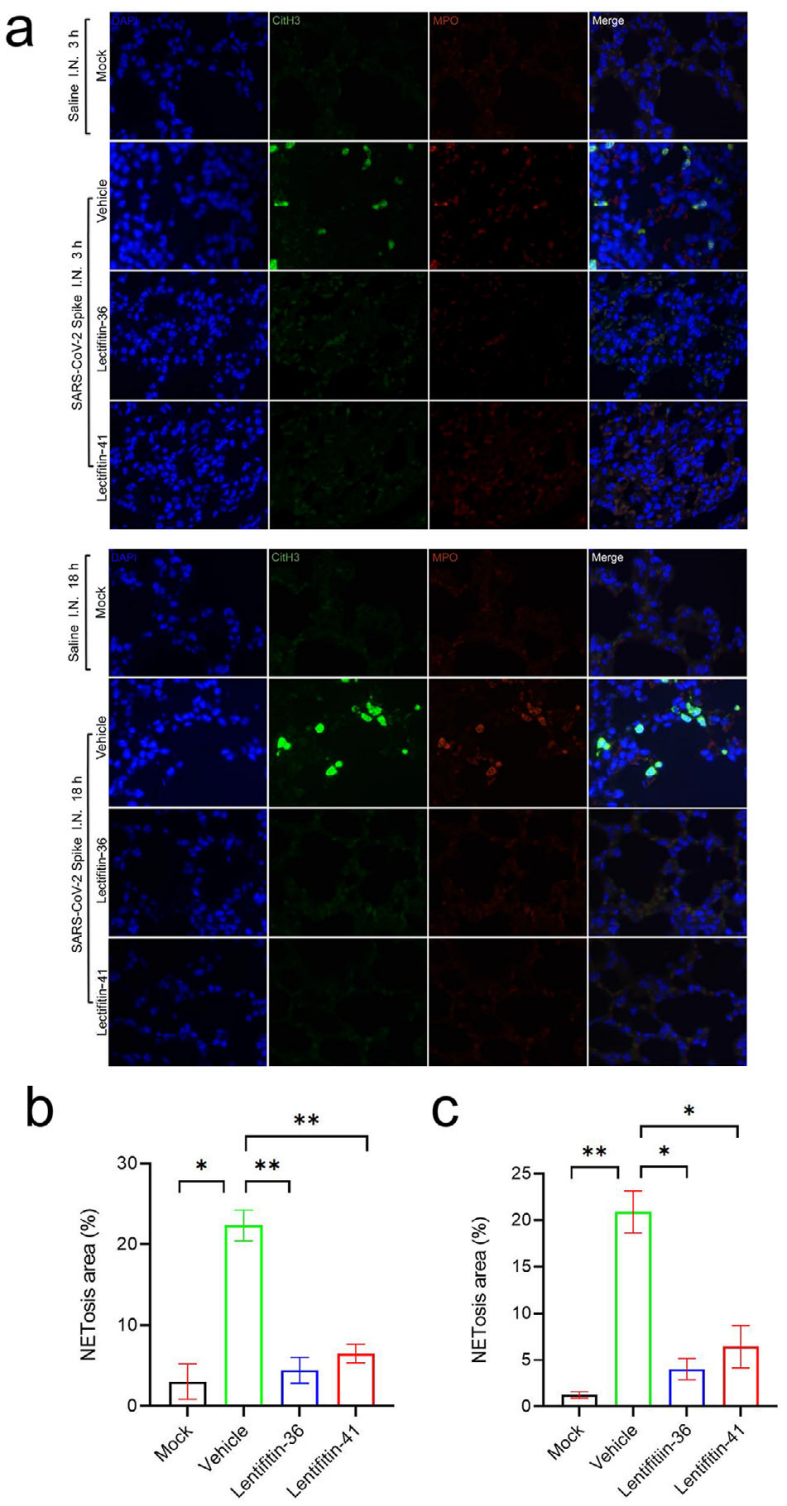
Inhibition of SARS‐CoV‐2 spike protein‐induced NETosis in lung tissue by Lectifitin‐36 and Lectifitin‐41.C57BL/6 mice were intranasally administered saline or SARS‐CoV‐2 spike protein. Lectifitin‐36/ Lectifitin‐41 (5 mg/kg) was intravenously injected 30 min prior to spike protein instillation, with a second dose administered 60 min post‐instillation. a) Confocal microscopy images of lung sections at 3 or 18 h post‐instillation show MPO (red) and CitH3 (green) co‐localization, with nuclei counterstained by DAPI (blue). Lectifitin‐36 and Lectifitin‐41 suppress NETosis in mouse lung tissue at both 3 h b) and 18 h c) after SARS‐CoV‐2 spike protein challenge. Representative quantification of MPO⁺/CitH3⁺ double‐positive cells (NET‐positive) was performed in lung sections at 3 and 18 h post‐instillation. NETosis area (%) was calculated from five randomly selected fields per mouse. Images represent one of three independent experiments; for each sample, five randomly selected fields were imaged. Scale bar 100 µm. Data are shown as mean ± SEM ^*^ *P* < 0.05, ^**^ *P* < 0.01 (unpaired Student's t‐test).

Previous studies indicate that the S protein induces NETosis in neutrophils, potentially exacerbating inflammation.^[^ ^20^ ^]^ To investigate whether the SARS‐CoV‐2 spike (S) protein induces neutrophil extracellular trap (NET) formation (NETosis) in vivo, mice were intranasally administered SARS‐CoV‐2 S protein diluted in physiological saline. As a vehicle control, phosphate‐buffered saline (PBS) was used. Tail vein injections of PBS (vehicle) were administered at 30 min prior to and 60 min after intranasal S protein administration. Lung tissues were harvested at 3 and 18 h post‐intranasal administration to assess NET markers, including myeloperoxidase (MPO) and citrullinated histone H3 (Cit‐H3), through immunostaining. Saline‐treated mice served as controls (Mock group) (**Figure** **4**).

As shown in Figure 4, abundant NET formation, characterized by the colocalization of MPO and Cit‐H3, was observed in the lungs of vehicle‐treated mice. These findings confirm that the SARS‐CoV‐2 spike protein can induce neutrophil NETosis, potentially contributing to the exacerbation of inflammatory responses. In contrast, mice treated with Lectifitin‐36/Lectifitin‐41 (5 mg kg^−1^) prior to and after S protein instillation displayed significantly reduced NET formation, as evidenced by decreased MPO and CitH3 co‐localization. These results demonstrate that ALPs effectively inhibit S protein‐induced NETosis in vivo, mitigating inflammatory responses.

### 2.5 Lectifitin‐36/Lectifitin‐41 Suppresses SARS‐CoV‐2 Spike‐Induced Lung Inflammation

**Figure 5:**
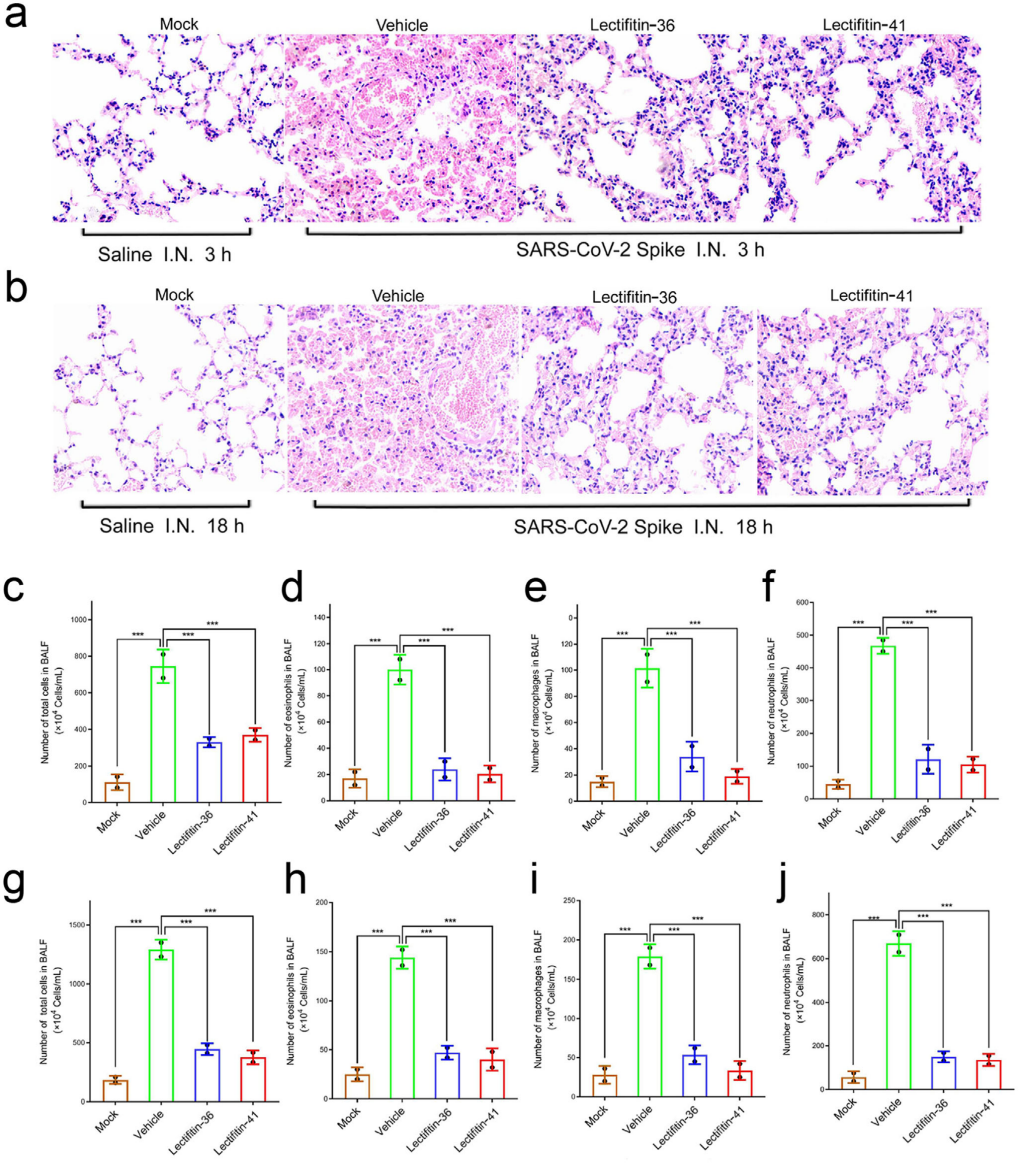
Inhibition of SARS‐CoV‐2 spike protein‐induced lung inflammation by Lectifitin‐36 and Lectifitin‐41.C57BL/6 mice were intranasally administered saline or SARS‐CoV‐2 S protein. Lectifitin‐36/Lectifitin‐41 (5 mg kg^−1^) was intravenously injected 30 min before and 60 min after S protein administration. Lung tissues were harvested at 3 h a) and 18 h b) post‐instillation and subjected to H&E staining for histological evaluation. Images represent one of three independent experiments; for each sample, five randomly selected fields were imaged. Scale bar 100 µm. c‐j) Lectifitin‐36/Lectifitin‐41 suppressed SARS‐CoV‐2 spike protein‐induced lung immunopathology. C57BL/6 mice were intranasally administered saline or SARS‐CoV‐2 spike protein. Lectifitin‐36/ Lectifitin‐41 (5 mg kg^−1^) was intravenously injected 30 min before and 60 min after spike protein administration. c,g) Total cell counts in BALF; d,h) Eosinophil counts in BALF; e,i) Macrophage counts in BALF; f,j) Neutrophil counts in BALF. Each bar represents mean ± SEM (*n* = 3). Statistical significance was analyzed using unpaired Student's t‐test: ^*^ *P* < 0.05; ^**^ *P* < 0.01; ^***^ *P* < 0.001; ^****^ *P* < 0.0001.

Previous studies have shown that the SARS‐CoV‐2 spike (S) protein induces excessive immune cell infiltration in vivo, driving severe inflammatory responses. To confirm this, SARS‐CoV‐2 S protein was diluted in saline and administered intranasally to C57BL/6 mice. Mice received intravenous injections of PBS (vehicle) 30 min before and 60 min after intranasal S protein administration. Mice in the mock group were treated with saline only. Lung tissues were harvested 3 and 18 h post‐instillation and subjected to histopathological examination using hematoxylin and eosin (H&E) staining.(**Figure** **5**a,b)

To evaluate the protective effects of Lectifitin‐36/Lectifitin‐41 against SARS‐CoV‐2 spike protein‐induced lung inflammation, broncho‐alveolar lavage fluid (BALF) was collected from C57BL/6 mice via tracheal cannulation and lung washing with PBS. The collected BALF was centrifuged, and the pellet was resuspended in PBS. Differential cell counts for eosinophils, macrophages, and neutrophils were determined based on cell size, morphology, and nuclear characteristics (Figure 5c–j).

As shown in Figure 5, vehicle‐treated mice administered with the SARS‐CoV‐2 spike protein exhibited significant inflammatory cell infiltration at both 3 and 18 h post‐instillation compared to mock‐treated mice. Total inflammatory cell counts in BALF, as well as the numbers of eosinophils, macrophages, and neutrophils, were markedly elevated, demonstrating that the spike protein acts as a potent inflammatory cell chemoattractant. In contrast, mice treated with Lectifitin‐36/Lectifitin‐41 exhibited significantly reduced total inflammatory cell counts and decreased numbers of eosinophils, macrophages, and neutrophils in BALF. These findings indicate that Lectifitin‐36/Lectifitin‐41 effectively alleviates inflammation in a mouse model of spike protein‐induced lung injury.

### 2.6 Lectifitin‐36/Lectifitin‐41 Reduces Immune Cell Recruitment and Vascular Damage in Lung Tissue

**Figure 6:**
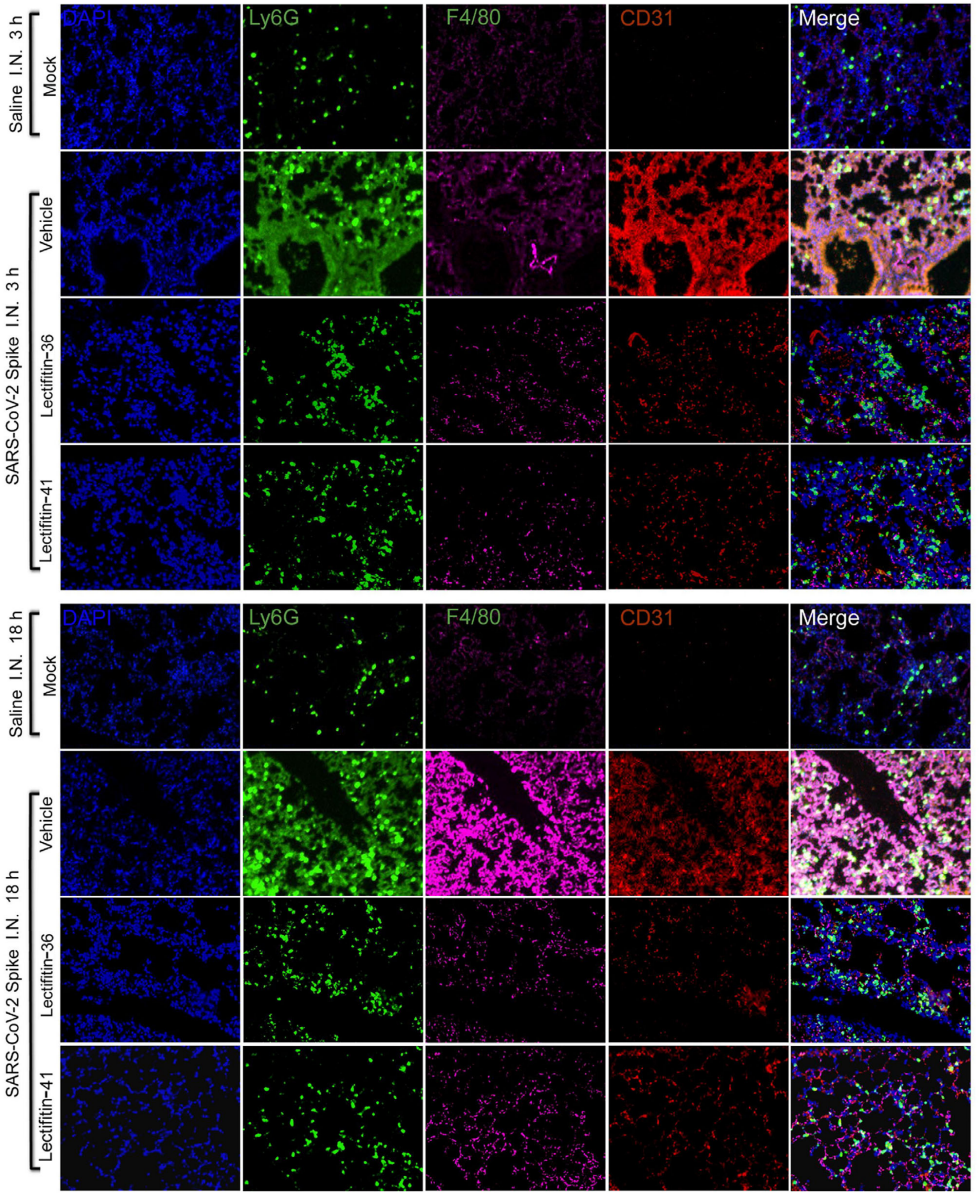
Inhibition of SARS‐CoV‐2 spike protein‐induced immune cell recruitment and vascular inflammation in lung tissue by Lectifitin‐36 and Lectifitin‐41. C57BL/6 mice were intranasally administered saline or SARS‐CoV‐2 S protein. Lectifitin‐36/Lectifitin‐41 (5 mg kg^−1^) was intravenously injected 30 min before and 60 min after S protein instillation. Confocal microscopy images show endothelial cells (CD31, red), alveolar macrophages (F4/80, purple), and neutrophils (Ly6G, green) in lung tissue 3 and 18 h post‐instillation. Images represent one of three independent experiments; for each sample, five randomly selected fields were imaged. Scale bar 100 µm.

C‐type lectins such as CLEC5A, CLEC6A, CD206, CLEC2, CLEC10A, DC‐SIGN, and L‐SIGN are highly expressed on neutrophils, monocytes, macrophages, dendritic cells, and platelets, and play critical roles in virus‐induced inflammation.^[^ ^18^ ^]^ To evaluate whether the S protein recruits inflammatory cells through its interaction with C‐type lectins, we performed immunofluorescence staining of lung tissue sections harvested at 3 h and 18 h post‐S protein administration. CD31 (red) was used to mark endothelial cells, F4/80 (purple) to label alveolar macrophages, and Ly6G (green) to identify neutrophils (**Figure** **6**).

As shown in Figure 6, vehicle‐treated mice exhibited increased vascular permeability, macrophage infiltration, neutrophil recruitment, and lung damage compared to mock‐treated mice. These changes were indicative of severe inflammation and epithelial cell detachment. To investigate whether Lectifitin‐36/Lectifitin‐41 could block the interaction between the S protein and C‐type lectins, thereby reducing inflammatory cell recruitment and lung inflammation, mice were treated with Lectifitin‐36/Lectifitin‐41 (5 mg kg^−1^) intravenously 30 min before and 60 min after S protein instillation. Immunofluorescence staining revealed that Lectifitin‐36/Lectifitin‐41 treatment significantly decreased the number of endothelial cells (CD31+), alveolar macrophages (F4/80+), and neutrophils (Ly6G+) in lung tissues compared to vehicle‐treated mice. These results demonstrate that Lectifitin‐36/Lectifitin‐41 possesses potent anti‐inflammatory properties in vivo.

### 2.7 Lectifitin‐36/Lectifitin‐41 Inhibits SARS‐CoV‐2 Spike Protein‐Induced Cytokine and Chemokine Release

**Figure 7:**
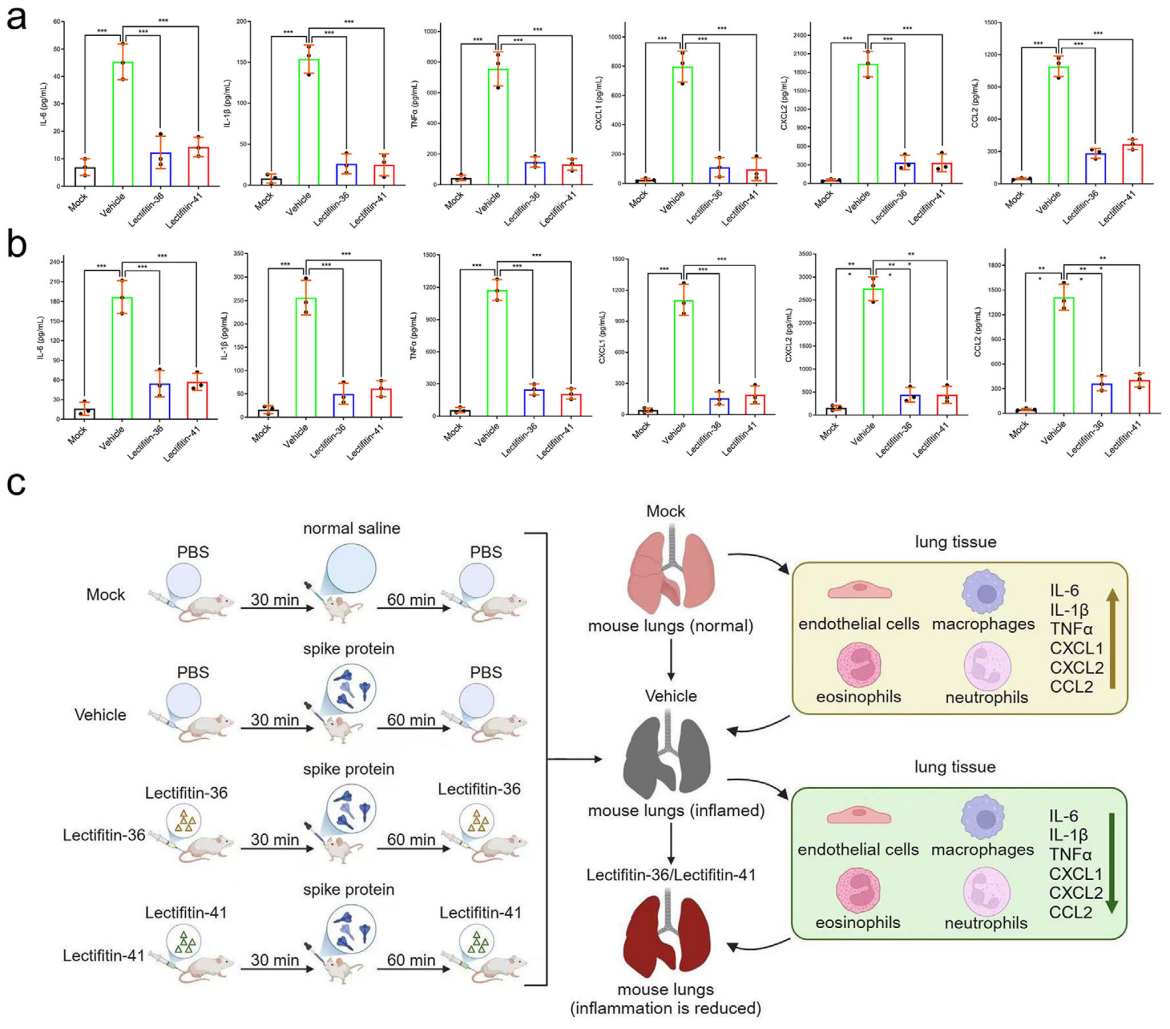
Inhibition of SARS‐CoV‐2 spike protein‐induced release of cytokines and chemokines in BALF by Lectifitin‐36 and Lectifitin‐41. C57BL/6 mice were intranasally administered saline or SARS‐CoV‐2 spike protein. Lectifitin‐36/Lectifitin‐41 (5 mg kg^−1^) was intravenously injected 30 min before and 60 min after spike protein administration. a) Cytokine and chemokine concentrations in BALF, including IL‐6, IL‐1β, TNF‐α, CXCL1, CXCL2, and CCL2, were measured via ELISA at 3 h. b) Cytokine and chemokine concentrations in BALF, including IL‐6, IL‐1β, TNF‐α, CXCL1, CXCL2, and CCL2, were measured via ELISA at 18 h. c) The figure describes the experimental design and outcomes of a mouse model investigating the effective therapeutic effect of Lectifitin‐36 and Lectifitin‐41 on inflammation induced by spike protein exposure. Inflammatory responses in the lungs and BALF are shown. Vehicle treatment led to increased inflammation, as evidenced by higher macrophage, eosinophil, and neutrophil counts, and elevated levels of IL‐6, IL‐1β, TNF‐α, CXCL1, CXCL2, and CCL2 in BALF. In contrast, Lectifitin‐36 and Lectifitin‐41 treatments reduced inflammation, as demonstrated by decreased macrophage and neutrophil counts, as well as lower concentrations of inflammatory cytokines (IL‐6, IL‐1β, TNF‐α) and chemokines (CXCL1, CXCL2, CCL2). Each bar represents mean ± SEM (*n* = 3). Statistical significance was analyzed using unpaired Student's t‐test: ^*^ *P* < 0.05; ^**^ *P* < 0.01; ^***^ *P* < 0.001; ^****^ *P* < 0.0001.

To investigate whether SARS‐CoV‐2 spike protein induces the release of inflammatory mediators such as cytokines and chemokines, BALF collected from mice was analyzed via ELISA for the levels of IL‐6, IL‐1β, TNF‐α, CXCL1, CXCL2, and CCL2. As shown in **Figure** **7**, vehicle‐treated mice exhibited significantly elevated levels of all measured cytokines and chemokines at 3 and 18 h post‐S protein administration compared to mock‐treated mice, indicating that the spike protein triggers robust inflammatory responses.

To determine whether Lectifitin‐36/Lectifitin‐41 could suppress this spike protein‐induced cytokine and chemokine release, Lectifitin‐36/Lectifitin‐41 was administered intravenously 30 min before and 60 min after spike protein instillation. Compared to vehicle‐treated mice, Lectifitin‐36/Lectifitin‐41‐treated groups displayed significantly reduced levels of IL‐6, IL‐1β, TNF‐α, CXCL1, CXCL2, and CCL2 in BALF. These results suggest that both Lectifitin‐36 and Lectifitin‐41 inhibited neutrophil infiltration and effectively suppress the release of pro‐inflammatory cytokines and chemokines, thereby mitigating spike protein‐induced inflammatory responses.

### 2.8 Lectifitin‐36/Lectifitin‐41 Exhibits Dose‐Dependent Neutralization of SARS‐CoV‐2 in Live‐Virus Infection Models

**Figure 8:**
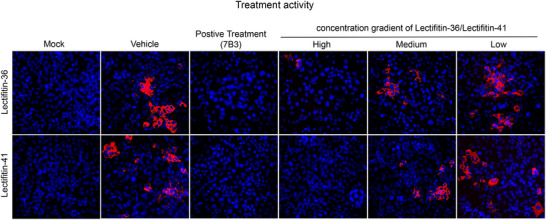
Neutralizing activity of Lectifitin‐36/Lectifitin‐41 against the SARS‐CoV‐2 BA.2 variant. Mock: Uninfected; Vehicle: Viral infection without treatment; Positive control: Virus pre‐incubation with 7B3 neutralizing antibody (10 µg mL^−1^), as described before;^[^ ^21^ ^]^ High/Medium/Low: Lectifitin‐36/Lectifitin‐41 (500 nm, 6.65 µg mL^−1^) at undiluted, 1:10, and 1:100 dilutions respectively. Immunofluorescence analysis of viral nucleocapsid (N) protein (red) was performed 24 h post‐infection. Nuclei were counterstained with DAPI (blue). Images represent one of three independent experiments; for each sample, five randomly selected fields were imaged. Scale bar 100 µm.

To validate the antiviral activity of Lectifitin‐36/Lectifitin‐41 against SARS‐CoV‐2, live virus neutralization assays were conducted using the Omicron BA.2 variant. Immunofluorescence analysis of the viral nucleocapsid (N) protein disclosed a dose‐dependent inhibitory effect in infected cell models (**Figure** **8**). The positive control 7B3 and high concentrations of Lectifitin‐41 achieved complete viral suppression. In contrast, even at the maximum concentration, Lectifitin‐36 permitted residual infection. Both Lectifitin‐36 and Lectifitin‐41 inhibited viral replication in a concentration‐dependent manner (5‐500 nm), with Lectifitin‐41 exhibiting significantly higher neutralizing potency than Lectifitin‐36.

## 3 Discussion

In this study, we developed two high‐affinity antibody‐like proteins (ALPs), Lectifitin‐36 and Lectifitin‐41, using cDNA display technology to target the SARS‐CoV‐2 spike protein. These ALPs effectively inhibit lectin pathway‐mediated complement activation, NETosis, and hyperinflammation in both in vitro and in vivo models, offering promising therapeutic potential against immune damage induced by the SARS‐CoV‐2 S protein.

Lectifitin‐36 and Lectifitin‐41 demonstrated strong nanomolar binding affinities for the spike protein, essential for blocking its interactions with ACE2^[^ ^22^ ^]^ and C‐type lectins. These interactions are critical in complement overactivation, excessive NET formation, and immune hyperactivation, which contribute to severe COVID‐19 pathology. Our results show that these ALPs effectively prevent the S protein from interacting with ACE2 and C‐type lectins, which are key mediators of immune cell activation and inflammation during infection.

MBL, a key recognition receptor of the innate immune system, activates the lectin pathway of complement upon binding to the S protein, contributing to tissue damage, particularly in the lungs. Inhibiting complement overactivation is thus a viable strategy to mitigate hyperinflammation and associated organ damage.^[^ ^6a,e^ ^]^ While other complement‐targeting therapies have been explored, this study introduces Lectifitin‐36 and Lectifitin‐41 as novel inhibitors of MBL‐S protein interactions, effectively blocking lectin pathway activation and reducing complement‐driven damage.

Additionally, neutrophils play a central role in SARS‐CoV‐2‐induced immune dysregulation. The S protein induces NET formation in neutrophils, exacerbating inflammatory responses and tissue damage. Lectifitin‐36 and Lectifitin‐41 could inhibit neutrophil activation through multiple mechanisms: by blocking RBD‐ACE2 interactions, preventing S protein binding to C‐type lectins, and suppressing lectin pathway activation. In vivo, treatment with Lectifitin‐36/Lectifitin‐41 significantly reduced NET formation and lung inflammation, confirming their potential to mitigate SARS‐CoV‐2‐induced immune damage.

Both in vitro and in vivo data show that Lectifitin‐36 and Lectifitin‐41 reduced inflammatory cytokine release, immune cell infiltration, and vascular damage, highlighting their ability to suppress inflammatory responses driven by S protein interactions with immune cells. Together, these findings position Lectifitin‐36 and Lectifitin‐41 as promising therapeutic candidates to address immune dysregulation and lung inflammation associated with COVID‐19. Furthermore, in our live‐virus assays, both Lectifitin‐36 and Lectifitin‐41 demonstrated effective dose‐dependent inhibition of SARS‐CoV‐2 replication. Notably, Lectifitin‐41 showed superior neutralizing potency, highlighting its potential as a more promising therapeutic candidate for combating SARS‐CoV‐2 infection.

Existing therapies typically target isolated components of the complement system or neutrophil's localized actions, without fully addressing the broad immune dysregulation mediated by the spike protein.^[^ ^23^ ^]^ In contrast, Lectifitin‐36 and Lectifitin‐41 offer a unique dual‐targeting strategy, simultaneously blocking both ACE2 mediated viral entry and C‐type lectin‐induced immune activation, providing a more comprehensive approach to modulating immune hyperactivation and mitigating tissue damage. This dual mechanism positions these ALPs as a novel and potentially more effective solution compared to traditional single‐target therapies, such as monoclonal antibodies targeting MASP‐2.^[^ ^10^ ^]^

In contrast to conventional neutralizing antibodies or anti‐complement agents, our ALPs exhibit a dual mechanism of action—concurrently blocking viral entry and attenuating immune hyperactivation. This integrated therapeutic strategy may provide broader and more cost‐effective protection, particularly in severe or immunopathology‐driven cases of COVID‐19.

This study developed Lectifitin‐36 and Lectifitin‐41, two high‐affinity antibody‐like proteins that block SARS‐CoV‐2 spike interactions with ACE2 and C‐type lectins. These ALPs effectively inhibit complement activation, NET formation, and hyperinflammation, reducing lung injury in vitro and in vivo. Their dual‐targeting mechanism offers a novel strategy for mitigating COVID‐19 immune dysregulation. Unlike traditional single‐target therapies, these ALPs provide a comprehensive approach to immune modulation. Future clinical validation is essential to assess their therapeutic potential in severe COVID‐19 and post‐acute complications.

## 4 Experimental Section

### Cell Culture

Human lung microvascular endothelial cells (HLMVEC, ATCC) and Vero E6 (ATCC) cells were maintained in DMEM supplemented with 10% fetal bovine serum (Sigma–Aldrich) at 37 °C under 5% CO_2_.

### Mice

C57BL/6 wild‐type (WT) mice (6–8 weeks old) were maintained under specific pathogen‐free (SPF) conditions in temperature‐controlled facilities with a 12 h light/dark cycle. Animals had ad libitum access to food and water. All procedures were approved by the Institutional Animal Care and Use Committee of Huazhong University of Science and Technology (Wuhan, China; approval no. 270193).

### Virus

The SARS‐CoV‐2 BA.2 variant (20220715) was provided by Hubei Provincial CDC. All infection experiments were conducted in biosafety level 3 (ABSL‐3) facilities with dual approval from Wuhan University and Hubei CDC.

### Plasmids, Transformation, and Expression

The gene encoding Lectifitin‐36/Lectifitin‐41 was cloned into a modified pQE‐80L vector containing an N‐terminal SUMO tag with an inserted thrombin cleavage site. Recombinant protein was expressed in *E. coli* BL21(DE3) cells (Beyotime). Bacteria were cultured at 37 °C in LB medium containing ampicillin. The temperature was reduced to 18 °C when OD_600_ reached 0.6. Expression was induced with 0.1 mm IPTG (isopropyl‐b‐D‐thiogalactopyranoside) and continued at 18 °C overnight. The cells were harvested by centrifugation and stored at −80 °C until use. The Lectifitin‐36 and Lectifitin‐41 were purified by Ni^2^⁺‐NTA affinity chromatography (Beyotime) in equilibration buffer (25 mm Tris‐HCl, pH 8.0, 150 mm NaCl) and eluted using a step gradient with 500 mm imidazole in the same buffer. Target protein liberation was achieved through thrombin cleavage (Beyotime, 1:2000) with subsequent 37 °C incubation for 6 h (Beyotime).

### Protein Interaction Analysis

Protein structures were predicted using AlphaFold3 (https://golgi.sandbox.google.com/) and visualized with PyMOL (version 3.1). The amino acid sequences of SARS‐CoV‐2 spike protein (UniProt: P0DTC2) were obtained from the UniProt database (https://www.uniprot.org/).

### ELISA

The SARS‐CoV‐2 receptor‐binding domain (RBD) and spike protein were obtained from Sino Biological (40592‐V08H, 40589‐V08B1). Binding activities of ALPs to SARS‐CoV‐2 spike protein were assessed via ELISA. spike protein (5 µg mL^−1^ in PBS) was coated onto 96‐well ELISA plates and incubated overnight at 4 °C. Plates were blocked with 200 µL of 2% BSA in TBST (10 mm Tris‐HCl, pH 7.5, 150 mm NaCl, 2 mm CaCl₂, 0.1% Tween‐20) at 37 °C for 2 h. Biotin‐labeled Lectifitin‐36, Lectifitin‐41, or BSA at varying concentrations was added and incubated for 1 h at 37 °C. After washing, HRP‐conjugated streptavidin (Biospa, 1:10000) was added and incubated for 1 h at 37 °C. Color was developed using TMB substrate (Thermo Fisher Scientific) and stopped with 2 M H₂SO₄. Absorbance was measured at 450 nm using a Varioskan ELISA plate reader (Thermo Fisher Scientific). EC_50_ values were determined using GraphPad Prism software.

### Competitive ELISA

Recombinant human ACE2, MBL, DC‐SIGN, L‐SIGN, CLEC5A, CLEC10A, and CLEC2 proteins (5 µg mL^−1^ in PBS; all purchased from Proteintech) were immobilized on 96‐well ELISA plates and incubated overnight at 4 °C. After blocking with 2% BSA in PBS, a fixed concentration of spike protein (5 µg mL^−1^) was pre‐incubated with increasing concentrations of biotinylated Lectifitin‐36, Lectifitin‐41, or BSA for 1 h at room temperature. The mixtures were then added to the coated wells. Following incubation and washing, bound biotinylated proteins were detected using HRP‐conjugated streptavidin, followed by standard substrate development and absorbance measurement as previously described. EC₅₀ values were calculated by nonlinear regression analysis using GraphPad Prism software.

### Surface Plasmon Resonance (SPR)

SPR was performed on a Biacore 8K instrument (GE Healthcare) at 25 °C. SARS‐CoV‐2 S protein was immobilized onto CM5 sensor chips via amine coupling. Lectifitin‐36 and Lectifitin‐41, prepared in Biacore running buffer, were flowed over the sensor chip at 10 µL min^−1^. Regeneration was performed with 10 mm glycine‐HCl, pH 2.5. Kinetic parameters were analyzed using Biacore Evaluation Software.

### Complement Deposition Assay

Spike protein (1 µg/mL in PBS) was coated onto an ELISA plate and incubated overnight at 4 °C. The wells were then blocked with BSA. Lectifitin‐36 and Lectifitin‐41, or BSA, were added and incubated at 37 °C for 1 h. Then 10% normal human serum (NHS; ComplementTech), heat‐inactivated NHS (HI‐NHS), MBL‐depleted serum, or MBL‐depleted serum supplemented with recombinant MBL were added and incubated at 37 °C for 1 h. After washing, rabbit anti‐C5b‐9 (ComplementTech), rabbit anti‐human C3a (Dako), and rabbit anti‐human C5a (Dako) antibodies were added and incubated at 37 °C for 1 h. HRP‐conjugated secondary antibody was added and incubated for 1 h, followed by three washes. TMB substrate was added, and absorbance was measured at 450 nm after 30 min.

### In Vitro Inflammatory Response Assay

Lectifitin‐36, Lectifitin‐41, BSA, and spike protein were diluted to a concentration of 1 µg mL^−1^ in PBS. The spike protein was then mixed with Lectifitin‐36, Lectifitin‐41, or BSA to generate the respective mixtures, with the spike protein/BSA mixture serving as the vehicle control. The mixtures were incubated at 37 °C for 1 h, with PBS serving as the mock control. Subsequently, 10% normal human serum (NHS), heat‐inactivated serum (HI‐NHS; 56 °C, 30 min), mannose‐binding lectin‐depleted serum (MBL ID), or MBL ID supplemented with recombinant MBL (MBL ID + rhMBL) was added and further incubated at 37 °C for 1 h. HLMVEC cells were seeded in 12‐well plates (1×10⁶ cells well^−1^) and incubated overnight. spike protein was mixed with Lectifitin‐36, Lectifitin‐41, or BSA and incubated with NHS, HI‐NHS, MBL‐depleted serum, or MBL‐reconstituted serum for 1 h. After a 4 h co‐culture, supernatants were collected and analyzed for VCAM‐1, ICAM‐1, and IL‐6 levels using ELISA kits (R&D Systems).

### Mouse Inflammation Model

C57BL/6 mice were randomly divided into 4 groups (*n* = 6 per group), including mock, vehicle, Lentifitin‐36, and Lentifitin‐41. Mice were intranasally instilled with 25 µg of S protein in 50 µL saline to establish a SARS‐CoV‐2 spike‐induced pneumonia model. Saline‐instilled mice served as mock controls. Mice were treated with intravenous Lectifitin‐36/Lectifitin‐41 (5 mg kg^−1^) 30 min before and 60 min after S protein instillation. Lung tissues were harvested at 3 and 18 h post‐instillation. Each experimental group included at least 3 biological replicates, and representative data from at least two independent experiments are shown.

### Histological Analysis

Lung tissues were fixed in 10% neutral‐buffered formalin overnight at room temperature, embedded in paraffin, and sectioned at 4 µm thickness. The sections were stained with hematoxylin and eosin (HE) following standard protocols. Stained slides were visualized for histological evaluation.

### Bronchoalveolar Lavage Fluid (BALF) Collection and Analysis

The lungs of mice were lavaged three times with 0.5 mL of sterile PBS to collect bronchoalveolar lavage fluid (BALF). The BALF was centrifuged at 300 × g for 10 min at 4 °C, and the supernatant was stored at −80 °C. Cytokine levels (IL‐6, IL‐1β, TNF‐α, CXCL1, CXCL2, and CCL2) were measured by ELISA (Thermo Fisher Scientific). The cell pellet was resuspended in PBS, and total cell count was determined using a hemocytometer. Differential cell counts were performed on Wright‐Giemsa stained slides.

### Neutralization Assay

Serial dilutions of Lectifitin‐36/Lectifitin‐41 were preincubated with viral particles (3.2 × 10⁶ PFU mL^−1^) in culture medium at 25 °C for 1 h before inoculation onto Vero E6 monolayers in 96‐well plates. The cell‐virus complexes were then incubated at 37 °C for 24 h prior to analysis. All experiments were independently repeated at least three times.

### Immunofluorescence Analysis

SARS‐CoV‐2‐infected Vero E6 cells were processed through PFA fixation, Triton X‐100 permeabilization, and sequential incubation with anti‐nucleocapsid antibody (Sino Biological, 40143‐MM05, 1:500) and Alexa Fluor 594‐conjugated secondary IgG (Thermo Fisher Scientific, A‐11032, 1:500). Nuclei were counterstained with DAPI (Beyotime, C1005). Fluorescence signals were documented via Leica DMi8 microscopy.^[^ ^24^ ^]^

### Immunofluorescence Triple Staining (TSA Method)

NETosis analysis: paraffin‐embedded lung sections underwent antigen retrieval in citrate buffer (pH 6.0) at 95 °C for 20 min, followed by sequential staining with anti‐MPO (Thermo Fisher Scientific, PA5‐16672, 1:200) and anti‐CitH3 (CST, 97272, 1:800) antibodies. TSA‐647 and TSA‐488 (Thermo Fisher Scientific, B40936, B40932) were used for TSA signal development. Antibody stripping was performed between cycles using 0.1 m glycine buffer (pH 2.5) for 10 min. Nuclei were counterstained with DAPI, and sections were mounted using antifade medium (Servicebio, G1401). Immune cell staining: sections were incubated with rabbit primary antibodies against CD31 (Abcam, ab182981, 1:100), F4/80 (CST, 70076, 1:100), and Ly6G (Abcam, ab238132, 1:100). Each primary antibody was followed by incubation with HRP‐conjugated goat anti‐rabbit IgG (Servicebio, GB23303, 1:500) for 30 min and TSA signal development using TSA‐647, TSA‐555 (Thermo Fisher Scientific, B40933), and TSA‐488.

### Statistical Analysis

Data preprocessing was performed using GraphPad Prism software. The sample size (n) and specific statistical methods used to test significant differences for each analysis were clearly indicated in the corresponding figure legends. Data were presented as mean ± SD and analyzed using Student's t‐test in SPSS 13.0. Differences with *P* < 0.05 were considered statistically significant.

## Conflict of Interest

The authors declare no conflict of interest.

## Author Contributions

Y.W. and C.B. contributed equally to this work. X.L. performed conceptualization. Y.W. and C.B. performed methodology. Z.Z. and M.G. performed validation. X.W. and K.H. performed investigation. X.L. C.B. wrote the original draft. X.L. and F.Y. performed supervision. X.L. and F.Y. acquired funding acquisition. All authors have read and agreed to the published version of the manuscript.

## Supporting information



Supporting Information

## Data Availability

The data supporting this study's findings are available with this article and its Supplementary Information or from the corresponding authors upon request.
