# Supplementary material for: Engineered Dual‐Function Antibody‐Like Proteins to Combat SARS‐CoV‐2‐Induced Immune Dysregulation and Inflammation
Source: Adv Sci (Weinh). 2025 Jul 6;12(37):e04690. doi: 10.1002/advs.202504690 (PMC12499391; doi:10.1002/advs.202504690)
Supplement: Supplementary file 1 — Supporting Information [file ADVS-12-e04690-s001.docx]

**Supplement Data**


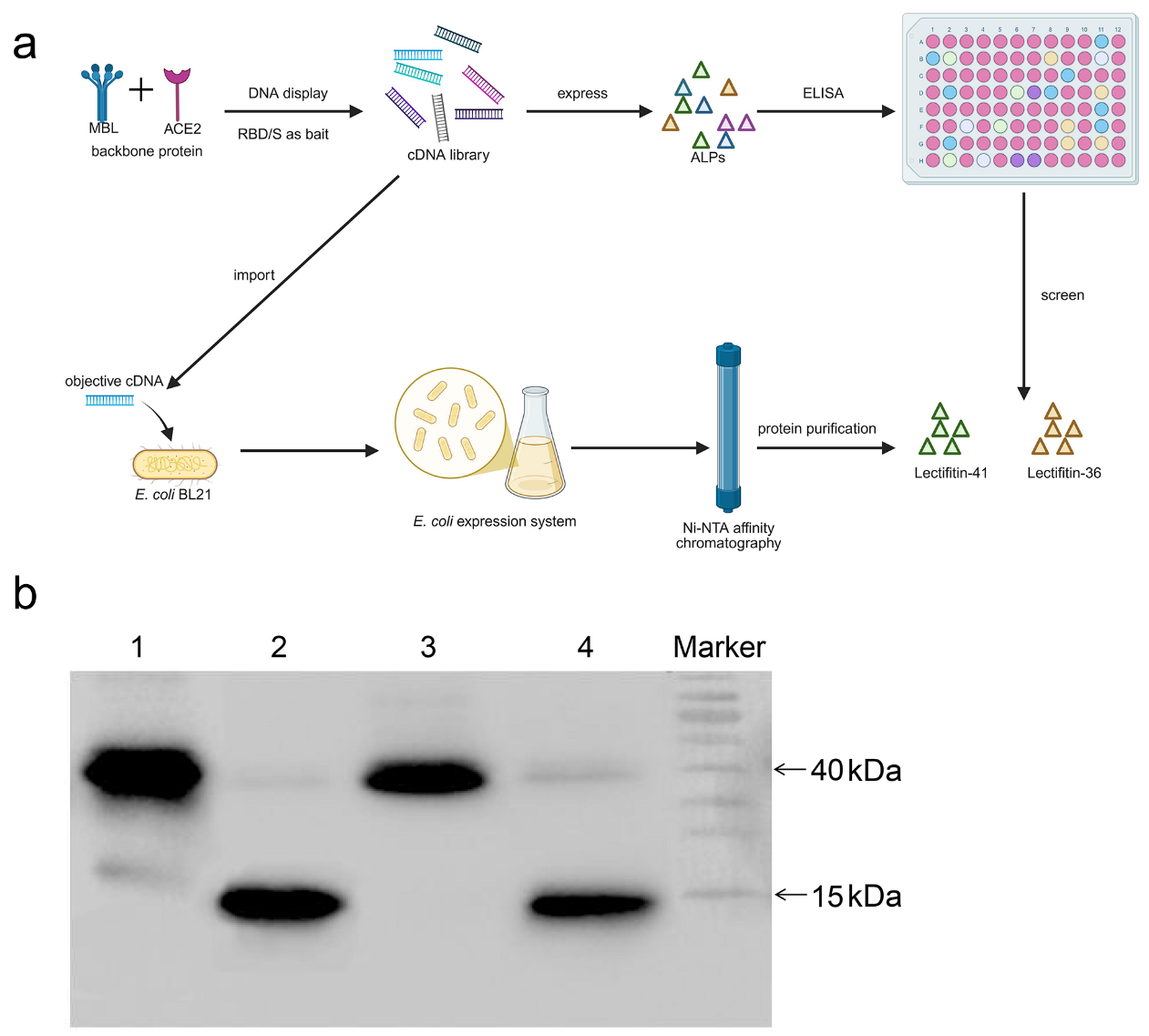


**Supplementary Figure 1.** Generation and Screening of Lectifitin-36/ Lectifitin-41 Targeting the SARS-CoV-2 Spike Protein. a) This diagram outlines the process for generating and screening antibody-like proteins (ALPs) that target the SARS-CoV-2 spike protein. A fusion protein of MBL and ACE2 is used as the backbone, with the spike protein's RBD as bait to screen a cDNA library. The selected ALPs are then expressed in *E. coli* BL21, purified via Ni-NTA affinity chromatography, and evaluated using ELISA to identify high-affinity candidates for further analysis. b) Western blot analysis. 1: Lectifitin-36 before protein enzyme digestion; 2: Lectifitin-36 after protein enzyme digestion; 3: Lectifitin-41 before protein enzyme digestion; 4: Lectifitin-41 after protein enzyme digestion. The full-length proteins migrated at approximately 40 kDa, while SUMO tag cleavage yielded products of 13 kDa.

**Experimental Section**

*cDNA Display of Lectifitins*: cDNA display was conducted following modified protocols. DNA templates were transcribed in vitro using the T7 RiboMAX™ system (Promega), and mRNA was purified (MicroElute Kit, OMEGA), quantified (EON reader), and analyzed by 1.5% agarose gel electrophoresis. The purified mRNA was ligated to a puromycin linker using T4 DNA ligase (NEB, #M0202) and validated by 7% urea-PAGE (8 M urea) with fluorescence imaging (Pharos FX, BioRad). The resulting conjugates were translated using the PURExpress system (NEB, #E6800/#E6820), and reverse transcribed with SuperScript II (Invitrogen) to yield mRNA/cDNA–protein complexes^[1].^ These complexes were incubated with biotinylated SARS-CoV-2 spike and RBD proteins immobilized on streptavidin M280 Dynabeads (ThermoFisher, #11205D) for affinity selection. After stringent washing (50 mM Tris-HCl pH 7.6, 1 mM EDTA, 500 mM NaCl, 0.1% Tween-20), bound cDNA was PCR-amplified and used for subsequent selection rounds[2].


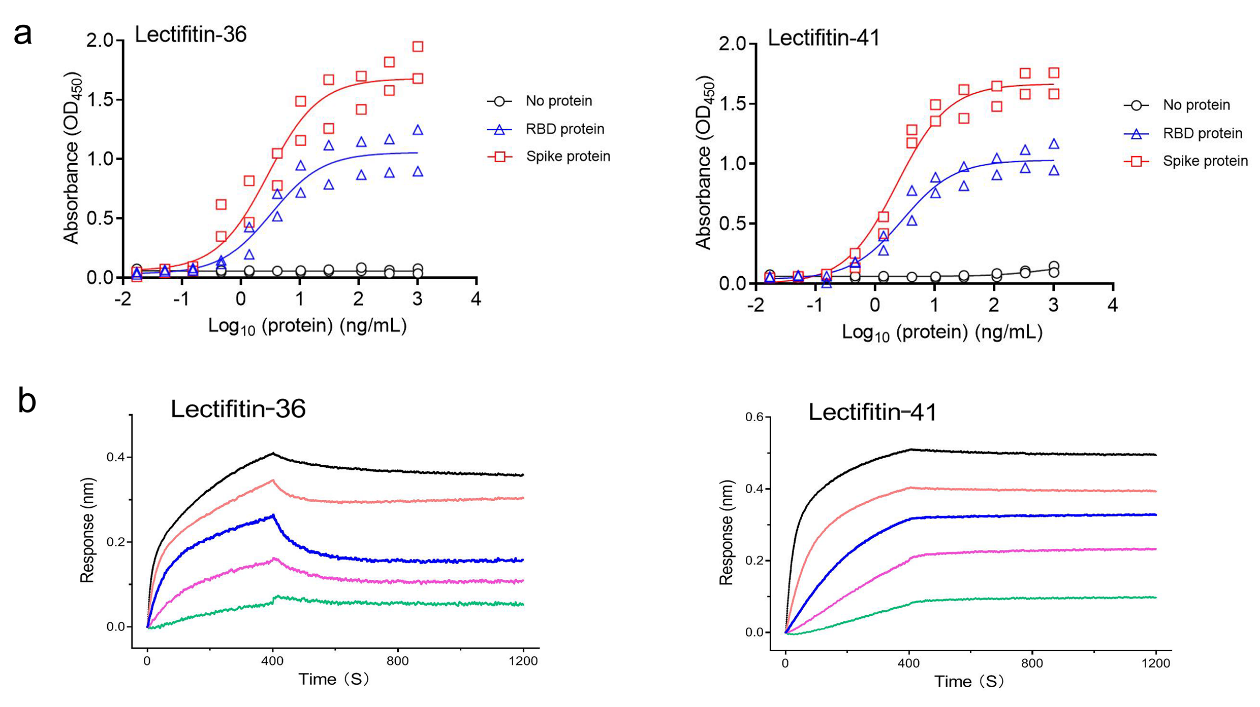


**Supplementary Figure 2.** Biochemical evaluation of Lectifitin-36/Lectifitin-41 targeting the SARS-CoV-2 S protein. a) ELISA dose-response curves illustrating the EC_50_ values for Lectifitin-36 and Lectifitin-41 binding to the RBD and S protein. Data represent mean ± SEM from two or three independent experiments. b) SPR analysis showing the binding kinetics and dissociation constants (Kᴰ) of Lectifitin-36 and Lectifitin-41 with the S protein.

**References**

[1] Y. Mochizuki, M. Biyani, S. Tsuji-Ueno, M. Suzuki, K. Nishigaki, Y. Husimi, N. Nemoto, *ACS Comb Sci* **2011**, *13* (5), 478, <https://doi.org/10.1021/co2000295>.

[2] a) R. W. Roberts, J. W. Szostak, *Proc Natl Acad Sci U S A* **1997**, *94* (23), 12297, <https://doi.org/10.1073/pnas.94.23.12297>; b) T. Kondo, Y. Iwatani, K. Matsuoka, T. Fujino, S. Umemoto, Y. Yokomaku, K. Ishizaki, S. Kito, T. Sezaki, G. Hayashi, H. Murakami, *Sci Adv* **2020**, *6* (42), abd3916, <https://doi.org/10.1126/sciadv.abd3916>.
